# Identification of immune-activated hematopoietic stem cells

**DOI:** 10.1038/s41375-018-0220-z

**Published:** 2018-07-24

**Authors:** Nir Bujanover, Oron Goldstein, Yariv Greenshpan, Hodaya Turgeman, Amit Klainberger, Ye’ela Scharff, Roi Gazit

**Affiliations:** 1 The Shraga Segal Department for Microbiology, Immunology and Genetics, Faculty of Health Sciences, Ben-Gurion University of the Negev, POB 84105 Beer-Sheva, Israel; 2 National Institute for Biotechnology in the Negev, Ben-Gurion University of the Negev, Beer-Sheva, Israel; 3 Center for Regenerative Medicine and Stem Cells, Ben-Gurion University of the Negev, Beer-Sheva, Israel

Hematopoietic stem cells (HSCs) are the source of both normal blood cells and leukemia [1]. The ability to identify and purify them using multiple surface markers, such as Lineage^-^cKit^+^Sca1^+^CD34^−^CD48^−^CD150^+^, facilitates the study of molecular mechanisms and dynamics of hematopoiesis [2, 3]. However, the identification of HSCs under immune-stimulation conditions poses a major challenge due to considerable changes in their pivotal markers, including Sca1 and CD150, following stimulation [4–10]. Therefore, we employ the *Fgd5*^mCherry^ reporter mouse to improve identification of activated HSCs. *Fgd5* is specifically expressed in HSCs of all hematopoietic cells, and the knocked-in *Fgd5*^mCherry^ reporter mouse was reported to specifically label HSCs under naive-state [11]. Here, we utilize *Fgd5*^mCherry^ to demonstrate the specific labeling of long-term multipotent cells under immune-stimulatory conditions, achieving better identification of activated-HSCs. RNA-Seq. data of purified Lineage^-^cKit^+^Sca1^+^CD150^+^Fgd5^mCherry+^ (LSK150^+^mC^+^) reveal molecular-changes in HSCs upon stress. We further identify CD317 (*Bst2)* and CD69 as new activation-markers of HSCs, demonstrating the response to stimulation at single-cell FACS resolution. This study thus presents the first mouse reporter for HSCs during immune stimulation, and highlights novel markers to monitor HSCs under stress conditions.

Using polyinosinic–polycytidylic acid (pIC), we stimulated a type-I interferon response in *Fgd5*^mCherry^ mice. We found that acute stimulation increased the expression of both Sca1 and CD150 (Fig. 1a, Supplementary Figure 1), in agreement with previous studies [5, 8, 9]. Importantly, 24 h post stimulation, the increase in Sca1 shifted almost the entire Lineage^-^cKit^+^Sca1^−^ (LK) population into the Lineage^−^cKit^+^Sca1^+^ (LSK) gate (Fig. 1a, b, Supplementary Figure 1). At 48 and 72 h post stimulation, the expression of both Sca1 and CD150 slowly reversed (Fig. 1b, Supplementary Figure 1). It is possible that part of the LK population was lost following stimulation, while many of these cells shift into the LSK-gate, which is in agreement with previous studies [5, 9]. Mobilization of the LSK population to peripheral-blood or spleen was not significant (data not shown). Dissection of the bone-marrow LSK compartment using CD150 and *Fgd5*^mCherry^ (mC) revealed that the frequency of LSK150^+^mC^−^ population, but not the LSK150^+^mC^+^ population, increased significantly (Fig. 1b top). Following an LPS stimulation, or an extended stimulation over 8 days, Sca1 and CD150 showed a similar increase of expression, while the frequency LSK150^+^mC^+^ population was unchanged (Supplementary Figures 2-3).

**Fig. 1:**
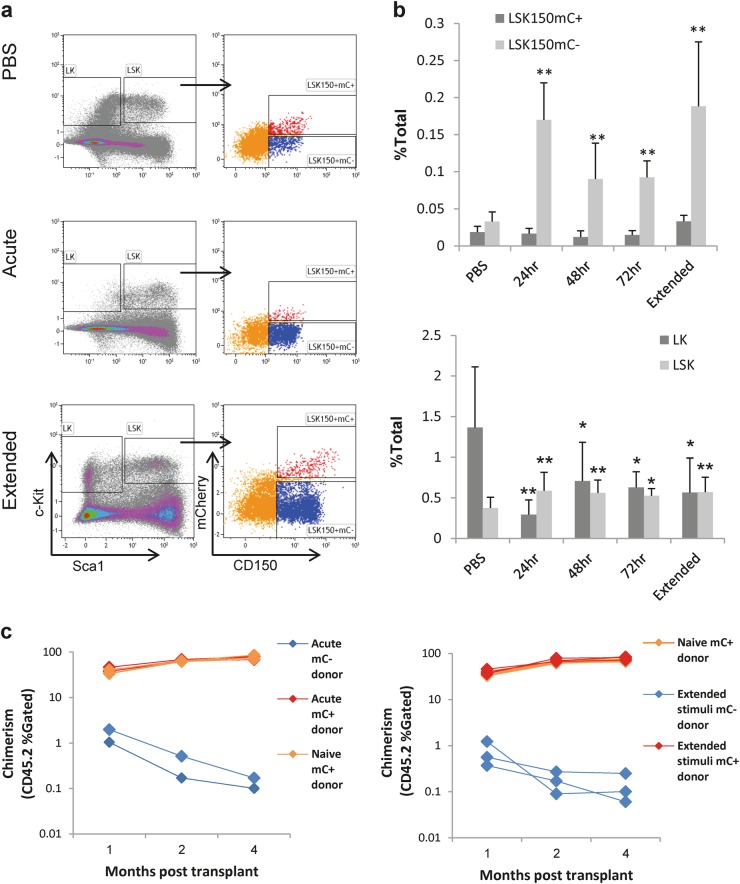
Acute and extended immune stimulation provokes conventional HSC markers. **a** Representative FACS plots, showing the staining of the Lineage^−^cKit^+^Sca1^+^ (LSK) compartment (left panels) and its dissection by CD150 and the *Fgd5*^mCherry^ reporter (mC, right panels) under control conditions (top) and under acute (middle) and extended (bottom) pIC stimulation, 24 h post stimulation. **b** Quantification of the frequencies of indicated cell populations: Lineage-cKit^+^Sca1^−^ (LK), Lineage^-^cKit^+^Sca1^+^ (LSK), LSKCD150^+^mC^−^, and LSKCD150^+^mC^+^. Histograms indicate mean frequency and standard deviation from the bone-marrow mononuclear cells (% of total). **c** LSKCD150^+^mC^+^ are functional HSCs. Chimerism (% of gated CD45.2 cells) over time following acute or extended pIC-stimulated donors. Data are from at least five mice per histogram; *p *<* 0.05, **p *<* 0.01

To functionally define this *Fgd5*^mCherry^ dissection of the LSK150 compartment, we transplanted either LSKCD150^+^mC^−^ or LSKCD150^+^mC^+^ cells together with congenic CD45.1 competitors into F1 recipients (Fig. 1c). This competitive-transplant enables three-way separation for competitor (45.1^+^45.2^−^), host (45.1^+^45.2^+^), and donor (45.1^–^45.2^+^), as shown in Supplementary Figure 4. Representative FACS plots (Supplementary Figures 4-7) demonstrate the analysis of test donor cells as myeloid Mac1^+^Gr1^−^ and Mac1^+^Gr1^+^ cells, or lymphoid CD3e^+^ T-cells and B220^+^ B-cells. Invariably, the LSK150^+^mC^+^ cells, and not the LSK150^+^mC^−^ cells, showed a robust multipotent long-term activity from either acute-stimulated or extended-stimulated bone-marrow cells (Fig. 1c, Supplementary Figures 4-7). Therefore, we gain better identification of HSCs under stress conditions. The LSKCD150^+^mC^−^ population showed relatively little expression of EPCR, which is important for repopulation-activity, and a low frequency of quiescence (data not shown).

After phenotypically and functionally identifying HSCs under immune-stimulation conditions, we continued to generate and analyze whole-transcriptome RNA-Seq data of LSKCD150^+^mC^+^ cells. Figure 2a shows differentially expressed genes of surface proteins of immune-stimulated HSCs, suggesting novel candidate markers for activated HSCs. Transcriptome data and RT-qPCR validation showed a pronounced increase in type-I and type-II interferons (IFNs; 163 out of the 435 significantly differentially expressed genes), a complex cell-cycle alteration; and a small change in HSC genes (16 out of 325), except for *Ly6a* (Sca1) and *MycN* (Supplementary Figure 8-9). The induction of cell-cycle progression under type-I IFN stimulation is not common, but not entirely unique to HSCs, as other immune cells, such as Th1 and NK cells, are known to proliferate upon IFN stimulation [12]. Intriguingly, the proliferation of HSCs is supported by the up-regulation of positive cell-cycle regulators for DNA synthesis, such as the origin recognition complex subunit 1 (*Orc1*), and a reduction in *Meg3*, which was recently shown to sustain HSC quiescence [13]. Conversely, upregulation of BTG anti-proliferation factor 3 (*Btg3*) and downregulation of the protein regulator of cytokinesis 1 (*Prc1*), are suggesting a genetic program to revert into quiescence already at the acute-activation stage, which is in agreement with previously published functional data [5].

**Fig. 2:**
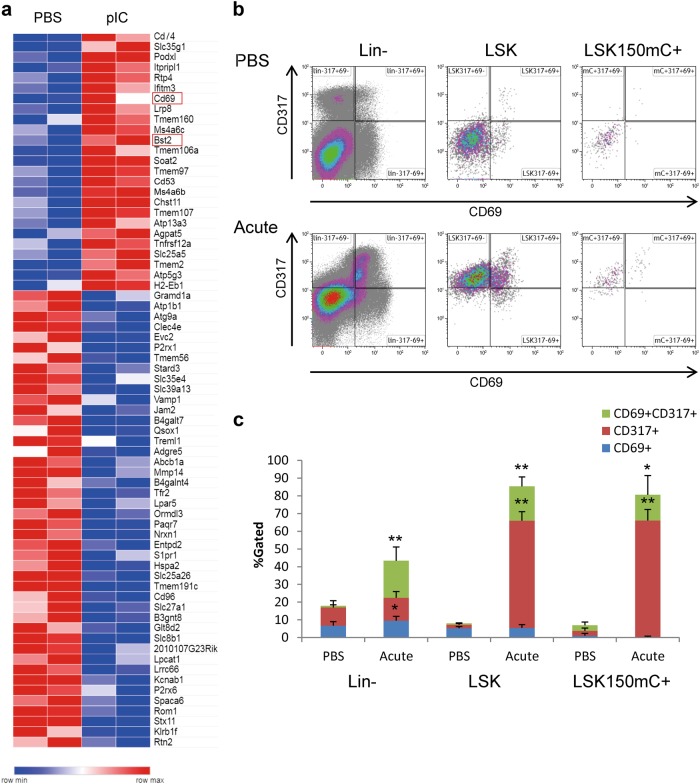
Surface markers of acutely activated HSCs. **a** A heatmap of 75 differentially expressed (DE) surface markers (GO:0016021, of 435 DE genes) under control (PBS) conditions and 24 h after an acute pIC stimulation. **b** Representative FACS plots showing the expression of CD69 (*x*-axis) and CD317 (*Bts2*, *y*-axis) in control conditions (PBS; top panels) and 24 h after an acute pIC stimulation (bottom panels). Plots are shown for the Lineage-negative (Lin^−^, left panels), Lineage^−^cKit^+^Sca1^+^ (LSK, middle panels), and LSKCD150^+^mCh^+^ (right panels) populations. **c** Quantification of the frequencies of indicated populations of CD69^+^CD317^+^ (green), CD317^+^CD69^−^ (red), and CD69^+^CD317^−^ (blue) cells. Histograms show averages and standard deviations of the control (PBS) and pIC-treated (Acute) cells; *p *<* 0.05, **p *<* 0.01

Candidate activation-markers includes *Clec2c* (CD69) and the bone marrow stromal cell antigen 2 (*Bst2*, also known as CD317). Importantly, expression changes may be uniform across all cells or heterogeneous at single-cell resolution. The latter alternative is of interest, as the HSC population is known to be functionally heterogeneous [14]. FACS analysis revealed an increase in CD317 and CD69 after acute pIC stimulation, confirming the RNA-Seq data to the protein level (Fig. 2b, c, Supplementary Figure 10). Notably, the increase in CD317 was uniform across all HSCs and across some of the early multipotent progenitors, suggesting that all of these cells responded to the IFN stimulation. In contrast, CD69 was only increased in part of the population, suggesting that some HSCs are more responsive to the stimulation than others. The identification of surface markers for HSC activation will enable the dynamic examination of the response and may pave the way towards functional examination of sub-populations of HSCs under stress.

Our data show that during immune stimulation, the majority of the LSK150^+^ population is non-HSC. Progenitor cells activities during infection will require further study, especially following their shift of expression of pivotal markers. This finding possesses a warning, as even an advanced single-cell analysis would confront the challenge of accounting for non-HSCs and could yield an ambiguous “novel classification” of HSC sub-populations. Our study demonstrates that *Fgd5*^mCherry^ reliably identifies stimulated HSCs, which retain transplantation potency following an acute stimulation; this notion is in agreement with the findings of Matatall et al. [7], who elegantly demonstrated that the reduction in HSCs and in repopulation activity was significant only after more than 2 months of *M. avium* infection.

It is yet to be determined whether HSCs play an essential physiological role in the inflammatory response, or whether they are activated as an innate response to an unknown threat. The CD317 expression pattern is providing the first direct evidence that all HSCs rapidly react to IFN, suggesting that the bone marrow niche is highly permeable in the context of immune stimulation. The observed heterogeneity in CD69 expression is in agreement with a functional heterogeneity among HSCs following infection [6], and suggests future functional assessment of HSC’s sub-populations. Interestingly, a very-recent study demonstrated potential mobilization and activation of HSCs and progenitors in a human-CD69 mouse model [15], suggesting further interest in this receptor. The identification of HSC-activation markers will enable the studies to refine the dynamic analysis of HSC during stress-induced hematopoiesis, which may lead to leukemia. The ability to better control our endogenous adult stem cells provides new opportunities to avoiding premature aging and a hazardous progression to pre-malignancy.

## Electronic supplementary material

Supplemental
