# Supplementary material for: Identification of immune-activated hematopoietic stem cells
Source: Leukemia. 2018 Jul 24;32(9):2016–20. doi: 10.1038/s41375-018-0220-z (PMC6127088; doi:10.1038/s41375-018-0220-z)

**Supplementary for manuscript "Identification of Immune Activated Hematopoietic Stem Cells", Bujanover et al.**

## Supplementary Methods:

### Mice and ethics

All mice were kept at the specific-pathogen-free unit of Ben-Gurion University of the Negev. Mouse strains used were *Fgd5*$^{mCherry}$ reporter, which has a knocked-in fluorescent-reporter mCherry in the Fgd5 gene, that is specifically expressed in HSCs of all hematopoietic cells[1]. *Fgd5*$^{mCherry}$ are on a C57Bl/6 background, having CD45.2 allotype. Congenic CD45.1 (Jax strain 2014) were used as competitors; F1 hybrids of CD45.1 × CD45.2, which express both surface markers, were used as hosts for competitive transplantation. All experiments were carried out according to the ethical committee guidelines following the approval of the local Ben-Gurion University of the Negev and Institutional Animal Care and Use Committees.

### Immune stimulation

Mice were stimulated intraperitoneally (IP) with 200 µg of a polyinosinic–polycytidylic acid (pIC, Sigma P1530). In a separate experiment, 20 µg of lipopolysaccharides of *E. coli* O55:B5 (LPS, Sigma L2880) were administered IP. Extended stimulation was performed by repeating this stimulation every other day over eight days in total.

### FACS

Cells from the bone marrow (BM) were extracted from the tibia, femur, and pelvis; sample media comprised phosphate-buffered saline (PBS) with 2 mM EDTA and 2% fetal calf serum (FCS). Mononuclear cells were enriched over histopaque (Sigma H1083) and stained as follows: Lineage: PacificBlue, Sca1: Apc, cKit: ApcCy7, and CD150: PeCy7 (Biolegend). Peripheral-blood samples were collected in Alsever's solution, underwent red blood cell lysis with an ammonium-chloride-potassium lysing buffer, and were then washed and stained as follows: CD45.2: PacificBlue, CD45.1: Apc, CD3e: PE, CD11b: PeCy7, B220: ApcCy7, Ter119: PerCPCy5.5, and Gr1: FITC (Biolegend). FACS Gallios

(Beckman-Coulter) and FacsAriaIII (BD) were used for analysis and sorting. Kaluza software was used to analyze FACS data.

**Transplantation**

HSCs were sorted as LSKCD150$^+$mC$^+$, mixed with competitor BM of CD45.1, and injected intravenously (IV) into lethally irradiated F1 recipients (Supplementary Figure 4a). This competitive transplantation settings allows for 3-way identification of the donor-cells (CD45.1-CD45.2+), residual host (CD45.1+CD45.2+), and competitor (CD45.1+CD45.2-). Peripheral blood was sampled 4, 8, 12, and 16 weeks after transplant.

**RNA-Seq**

HSCs were sorted by the phenotype of LSK150$^+$mC$^+$ from either control (PBS-injected) or immune-stimulated mice after pIC treatment (24 h post-injection). Cells were frozen in SMARTer buffer (1000 cells in 10.5 µl) and stored at -80 °C. RNA sequencing was performed at the Israel National Center for Personalized Medicine (INCPM, Rehovot), yielding some 20 million single-reads of 61 bases per sample. Files were analyzed using BaseSpace differential expression analysis v2.0.0 using TopHat2, Bowtie2, and cufflinks2. IFN signature was analyzed by the INTERFEROM web tool (http://www.interferome.org, v2.01) [2]. Gene lists were obtained from the Gene-Ontology database through MGI (http://www.informatics.jax.org/function.shtml).

## Supplementary References


1. Gazit R, Mandal PK, Ebina W, Ben-Zvi A, Nombela-Arrieta C, Silberstein LE, *et al.* Fgd5 identifies hematopoietic stem cells in the murine bone marrow. *The Journal of experimental medicine* 2014 Jun; **211**(7)**:** 1314-1330.

2. Rusinova I, Forster S, Yu S, Kannan A, Masse M, Cumming H, *et al.* Interferome v2.0: an updated database of annotated interferon-regulated genes. *Nucleic acids research* 2013 Jan; **41**(Database issue)**:** D1040-1046.

**Supplementary Figure 1: Identification of HSCs following acute pIC activation (24, 48, and 72 h post activation)**

**a** Experimental plan, showing the time of induction and analysis of HSCs. **b** Representative FACS plots for the staining of the $Lineage^{-}cKit^{+}Sca1^{+}$ (LSK) compartment (left panels) and its dissection by CD150 and the *Fgd5*$^{mCherry}$ reporter (mC , right panels) under control conditions (PBS, top panels) and after 24, 48, and 72 h of stimulation (lower panels). **c** Quantification of the indicated cell populations: Lineage-$cKit^{+}Sca1^{-}$ (LK), $Lineage^{-}cKit^{+}Sca1^{+}$ (LSK), $LSKCD150^{+}mC^{-}$, and $LSKCD150^{+}mC^{+}$. Histograms indicate mean frequency and bars show the standard deviation of the frequencies of each cell type from bone-marrow mononuclear cells (% of total). Data are from at least five mice per histogram; *$p < 0.05$, **$p < 0.01$.

**Supplementary Figure 2: Identification of HSCs following LPS stimulation**. **a** Representative FACS plots for the staining of the $Lineage^{-}cKit^{+}Sca1^{+}$ (LSK) compartment (left panels) and its dissection by CD150 and the *Fgd5*$^{mCherry}$ reporter (mC, right panels) under control conditions (PBS; top panel) and after 24, 48, and 72 h post LPS stimulation (lower panels). LPS stimulation provides TLR4 activation, unlike pIC activation of TLR3. **b,c** Quantification of the indicated cell populations: Lineage-$cKit^{+}Sca1^{-}$ (LK), $Lineage^{-}cKit^{+}Sca1^{+}$ (LSK), $LSKCD150^{+}mC^{-}$, and $LSKCD150^{+}mC^{+}$. Histograms indicate mean frequency and bars show standard deviation of each cell type from bone-marrow mononuclear cells (% of total). Data are from at least five mice per histogram; *$p < 0.05$, **$p < 0.01$.

**Supplementary Figure 3: Identification of HSCs following extended 1 week pIC activation**. **a** Experimental plan with indicated repeated pIC stimulations every other day and analysis thereafter. **b** Representative FACS plots for the staining of the $Lineage^{-}cKit^{+}Sca1^{+}$ (LSK) compartment and its dissection by CD150 and the *Fgd5*$^{mCherry}$ (mC) reporter under control conditions (PBS; top panel) or following an extended pIC stimulation (lower panels). **c** Quantification of the indicated cell

population: Lineage$^-$cKit$^+$Sca1$^-$ (LK), Lineage$^-$cKit$^+$Sca1$^+$ (LSK), LSK CD150$^+$mC$^-$, and LSKCD150$^+$mC$^+$. Histograms indicate mean frequency and bars show standard deviation of each cell type in bone-marrow mononuclear cells (% of total). Data are from at least three mice per histogram; *$p < 0.05$ and **$p < 0.01$.

**Supplementary Figure 4: *Fgd5*$^{\mathbf{mCherry}}$ labels long-term multipotent HSCs during acute immune stimulation. a** Experimental plan, indicating the stimulation of the donor mice followed by bone-marrow extraction and sorting for transplantation together with unstimulated bone marrow of CD45.1 congenic competitor cells. **b** Representative FACS plots of peripheral blood analysis of recipients at the long-term (16 weeks) time point. Left panels show the distinction of the Donor (CD45.2 on the x axis) from the Competitor (CD45.1, on the y axis) and the residual host (CD45.1+2). Examination of Donor cells for myeloid subtypes Mac1$^+$Gr1$^+$ and Mac1$^+$Gr1$^-$ is shown in the middle panels, and for lymphoid B-cells (B220$^+$) and T-cells (CD3e$^+$) on the right panels. **c-e** Quantification for the total chimerism (top), lineage types (middle), and chimerism over time (bottom), summarizing one of three independent experiments performed.

**Supplementary Figure 5: *Fgd5*$^{\mathbf{mCherry}}$ labels long-term multipotent HSCs during acute immune stimulation. a,b** Examination of Donor cells (chimerisem) for myeloid subtypes Mac1$^+$Gr1$^+$ and Mac1$^+$Gr1$^-$ and for lymphoid B-cells (B220$^+$) and T-cells (CD3e$^+$). **c** Quantification of the chimerism over time, summarizing one of three independent experiments performed. **d** Representative FACS plots for the peripheral blood analysis of recipients at the long-term (16 weeks) time point from control donors (PBS-treated LSK150mC+, donor HSCs).

**Supplementary Figure 6: *Fgd5*$^{\mathbf{mCherry}}$ labels HSCs after extended 1 week stimuli**. **a** Representative FACS plots for peripheral blood analysis of recipients at the long-term (16 weeks) time point. Left panels show the distinction of the Donor (CD45.2 on the x-axis) from the Competitor (CD45.1, on the y-axis) and the residual host (CD45.1+2). Dissection of Donor cells for myeloid subtypes Mac1$^+$Gr1$^+$ and

Mac1$^{+}$Gr1$^{-}$ is shown in the middle panels. Dissection for lymphoid B-cells (B220+) and T-cells (CD3+) is shown on the right panels. **b-d** Quantification for the total chimerism (top), lineage types (middle), and chimerism over time (bottom), summarizing one of three independent experiments performed.

**Supplementary Figure 7: *Fgd5*$^{\mathbf{mCherry}}$ labels HSCs after extended 1 week stimuli**. Dissection of the Donor cells for myeloid subtypes Mac1$^{+}$Gr1$^{+}$ and Mac1$^{+}$Gr1$^{-}$ is shown in the middle panels. Dissection of lymphoid B-cells (B220$^{+}$) and T-cells (CD3$^{+}$) is shown on the right panels. **a-c** Quantification of the total chimerism (top), lineage types (middle), and chimerism over time (bottom), summarizing one of three independent experiments performed.

**Supplementary Figure 8: Transcriptome analysis of immune-stimulated HSCs reveals a strong type-I/II IFN signature and cell cycle activation**. **a** Differential expression of genes in naïve controls and pIC-stimulated HSCs (LSK150$^{+}$mC$^{+}$, at 24 h following stimulation), analyzed for interferon response; numbers of genes associated with each IFN signature is summarized in the Venn diagram and their expression profile is shown (up-regulated, green arrow; down-regulated, red arrow). The heatmap presents 61 genes that are part of both the type-I and the type-II IFN signatures. Relative expression is represented from high (red) to low (blue). **b** Cell cycle annotated differentially expressed genes: “Positive” regulators are labeled as green squares; “negative” regulators are labeled as red squares. **c** HSC genes that changed following stimulation. Differentially expressed genes were calculated on FPKM values with a cutoff of $p < 0.11$ and a fold-log change > 0.9.

**Supplementary Figure 9: RT-qPCR validation of differentially expressed genes.** Quantification of nine differentially expressed E genes: *CD74*, *Cxcl10*, *Ifit1*, *Oas2*, *Oas3*, *Ifitm3*, *Camp*, *S100a8*, and *S100a9*. Total RNA was extracted from LSK150mC$^{+}$ cells of control and acutely treated mice (PBS and pIC, respectively). The histograms show mean and standard deviation of a quadruplet sampled RT-qPCR run.

**Supplementary Figure 10: CD69 and CD317 activation markers on stem and progenitor cells**. Representative FACS plots for the expression of CD69 (x axis) and CD317 (*Bts2*, y axis) under control conditions (PBS; top panels) or 24 h after pIC stimulation (bottom panels).

# Supplementary 1: pIC acute activation 24,48 and 72 hrs. post-activation

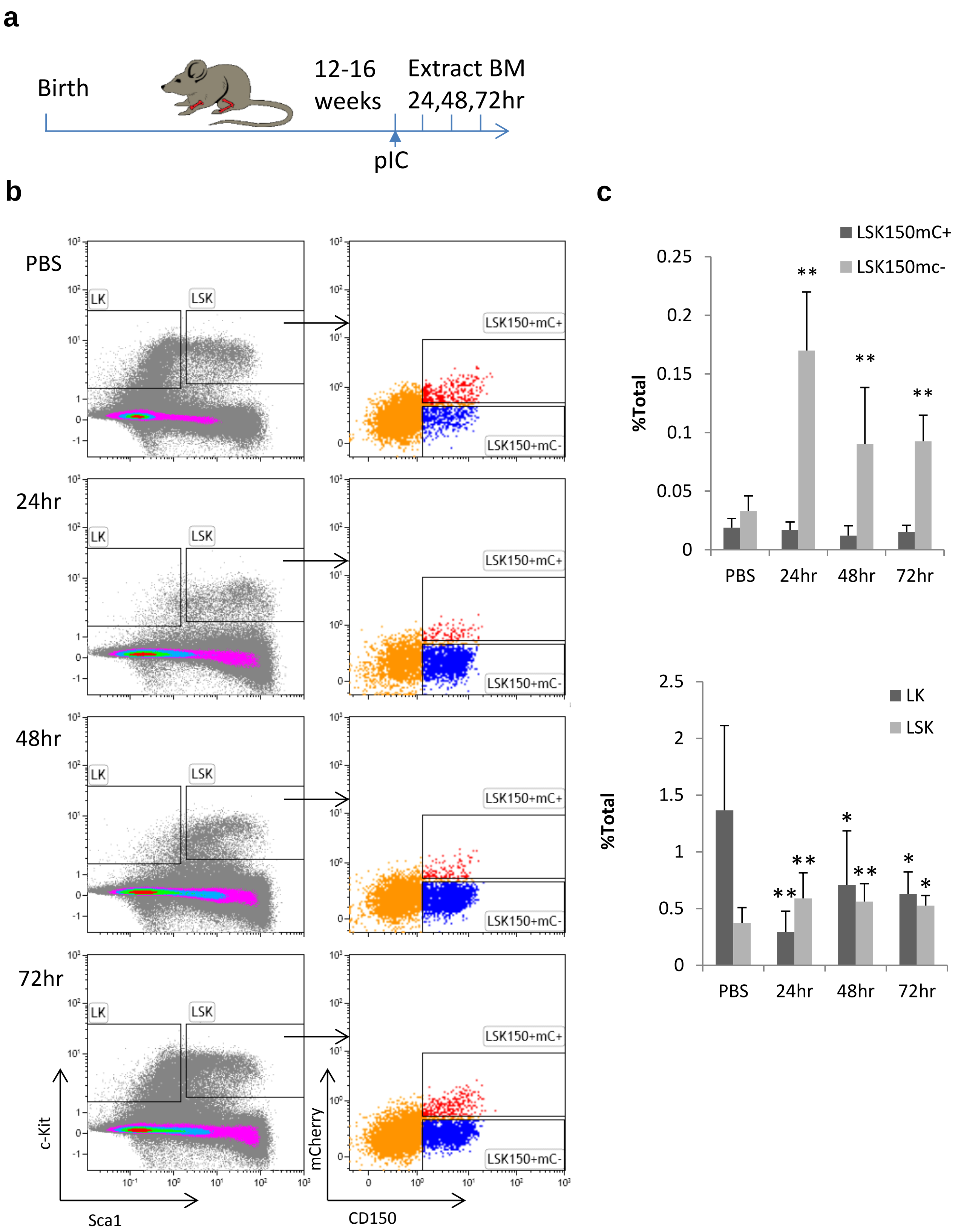

# Supplementary 2: LPS stimulation

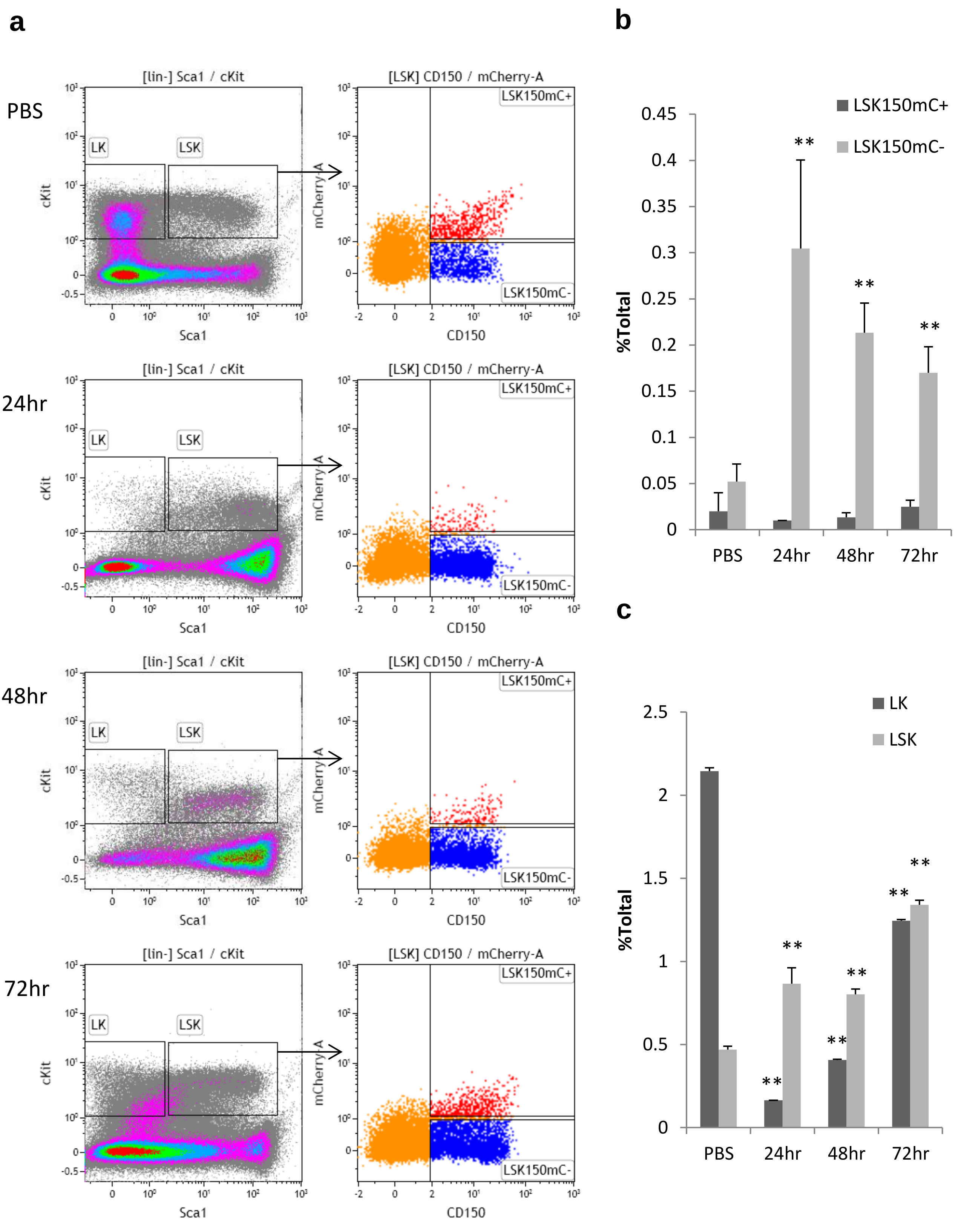

# Supplementary 3: extended pIC activation

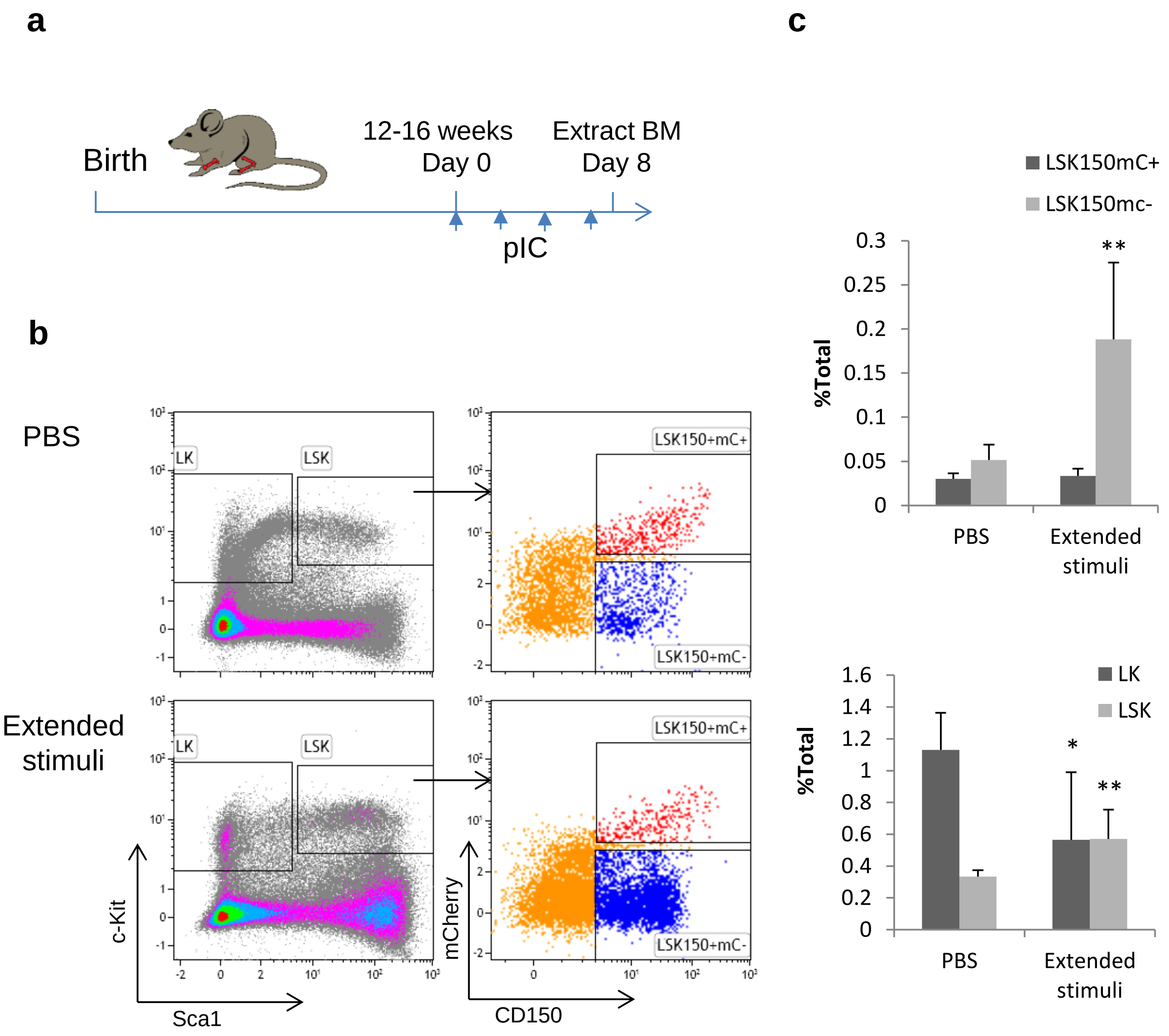

Supplementary 4: *Fgd5*$^{mCherry}$ labels long-term multipotent HSCs during acute immune stimulation

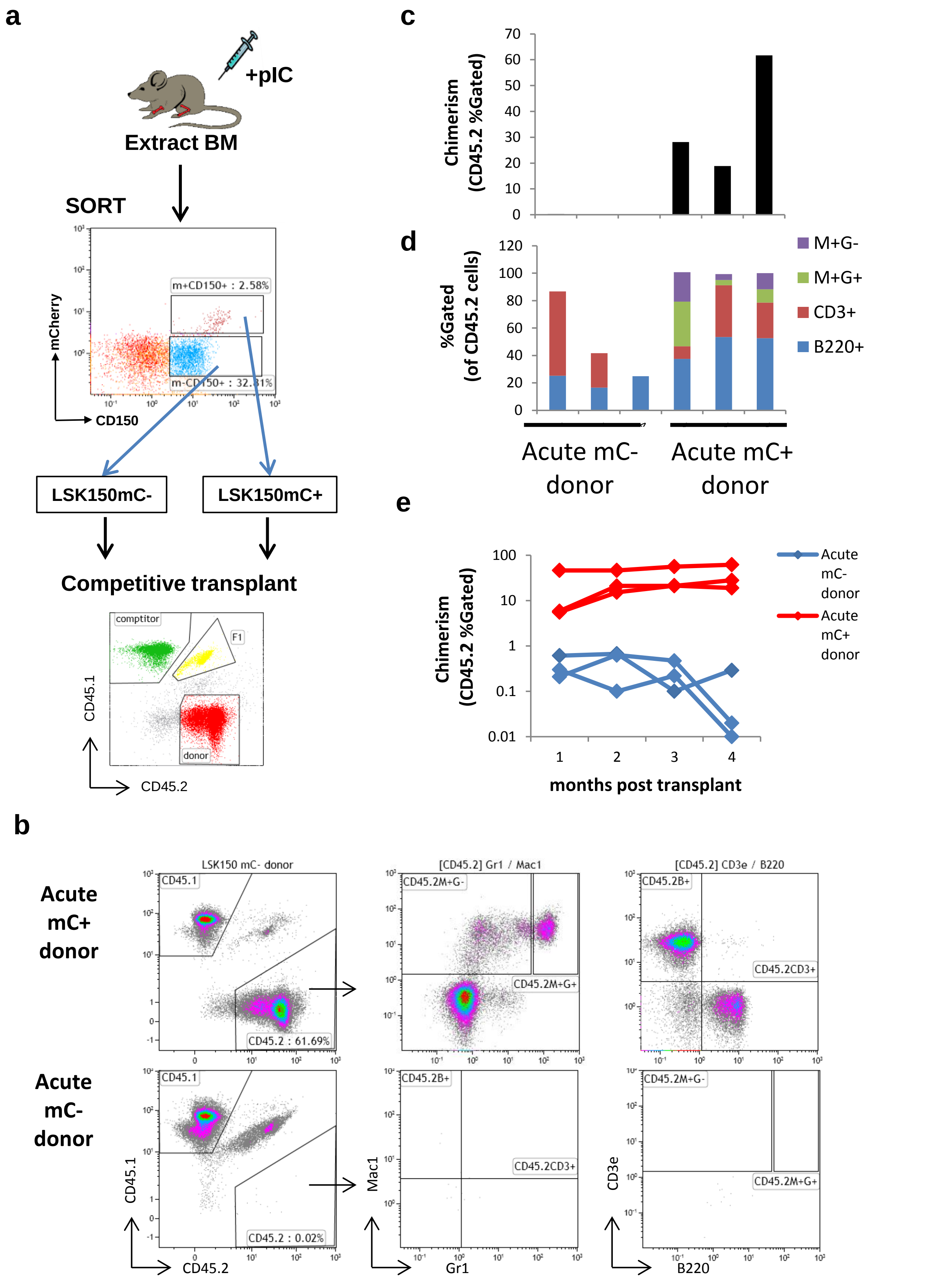

# Supplementary 5: *Fgd5*$^{mCherry}$ labels long-term multipotent HSCsduring acute immune stimulation

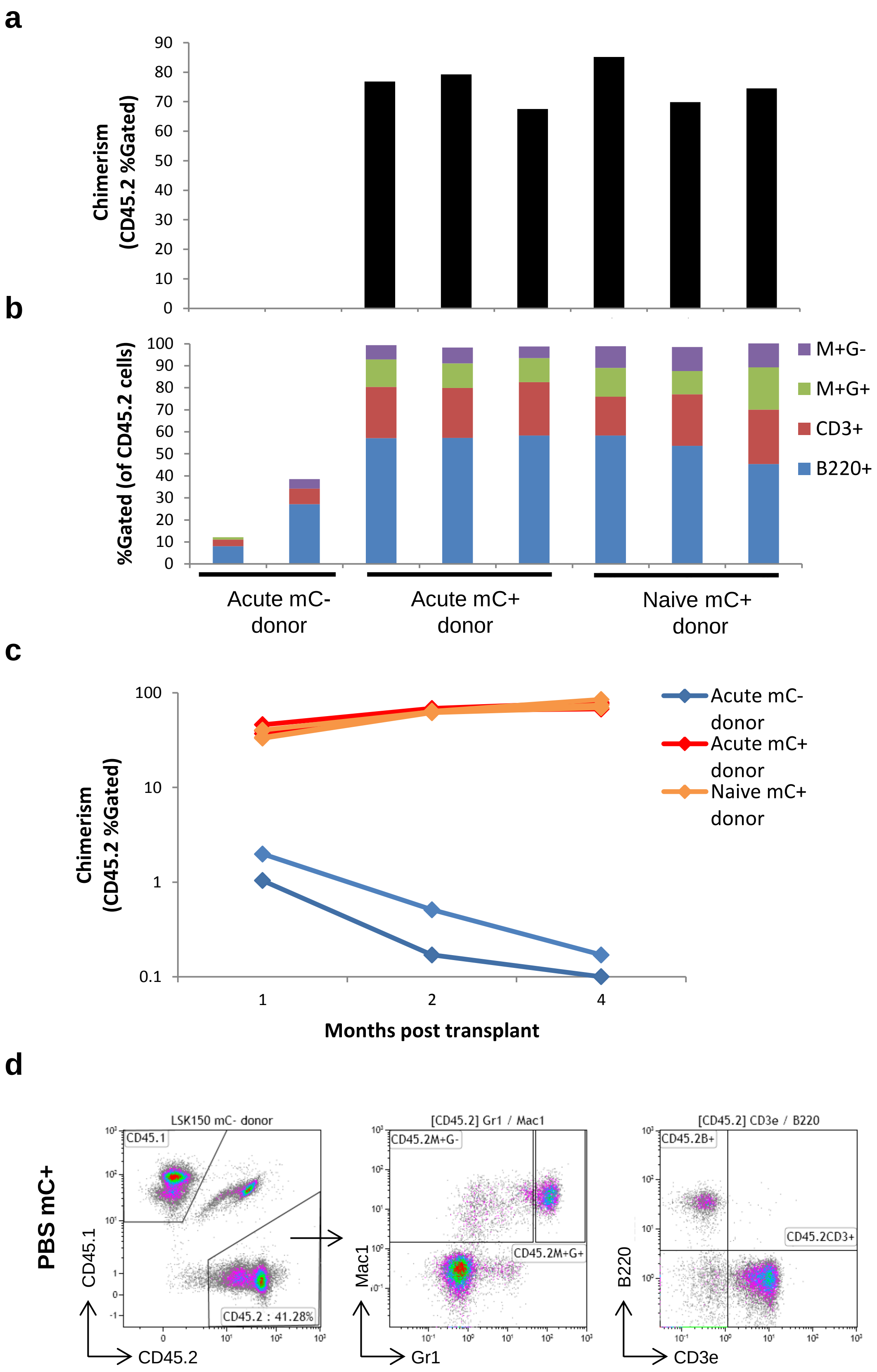

Supplementary 6: *Fgd5*$^{mCherry}$ labels HSCs after extended stimuli

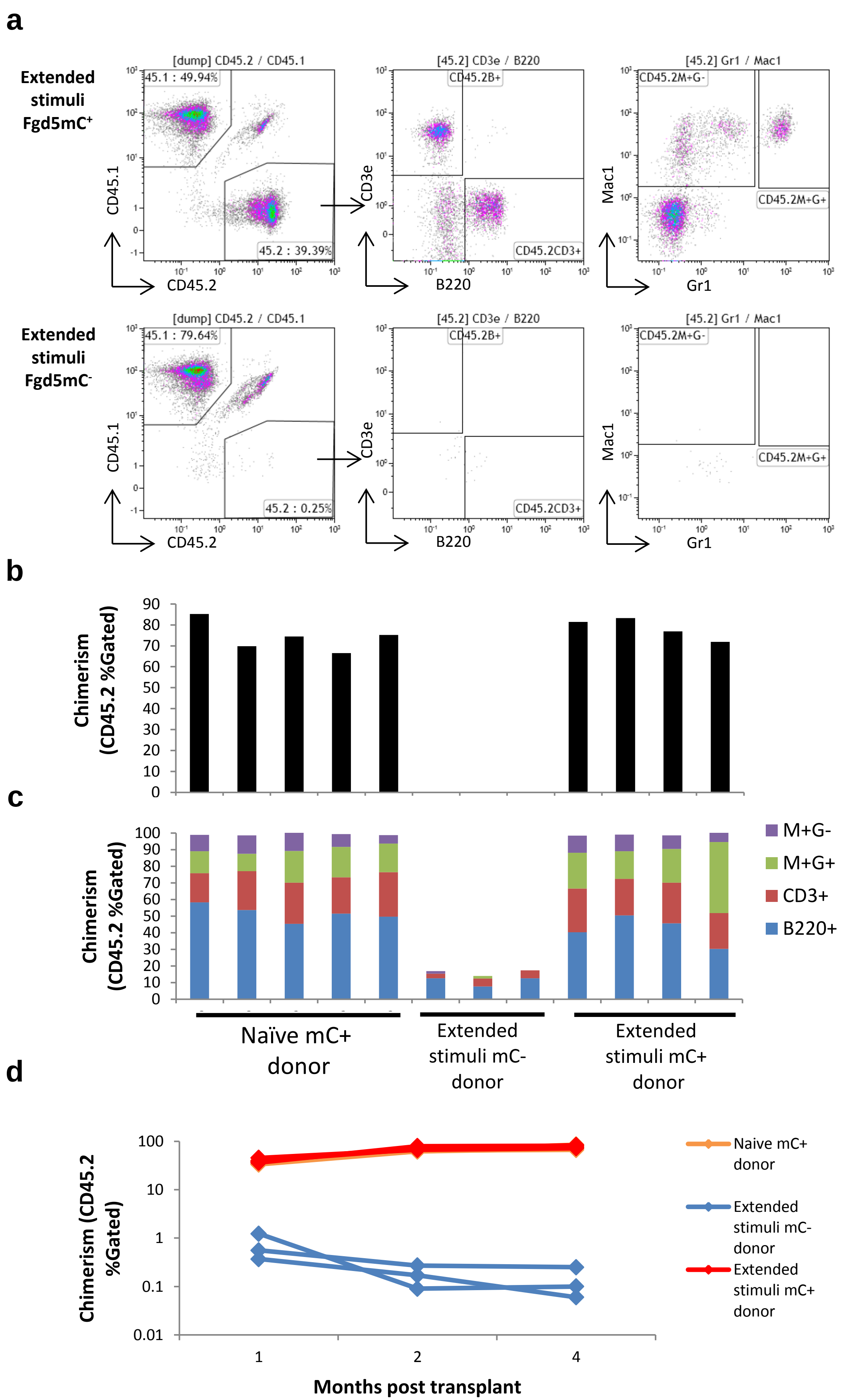

# Supplementary 7: Fgd5 labels HSCs after extended stimuli

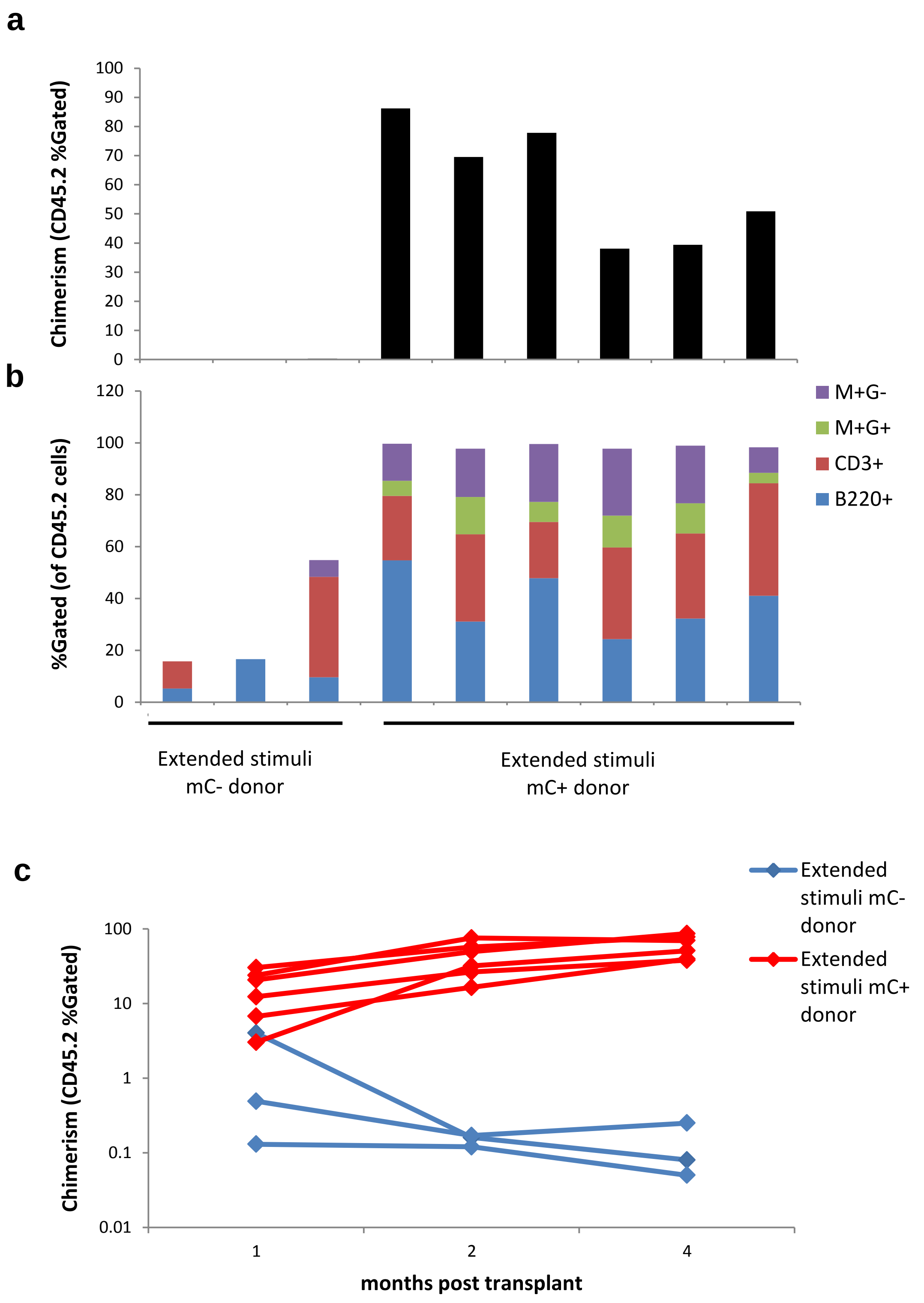

# Supplementary 8: Transcriptome analysis of immune-stimulated HSCs reveals a strong type-I/II IFN signature and activation of cell-cycle

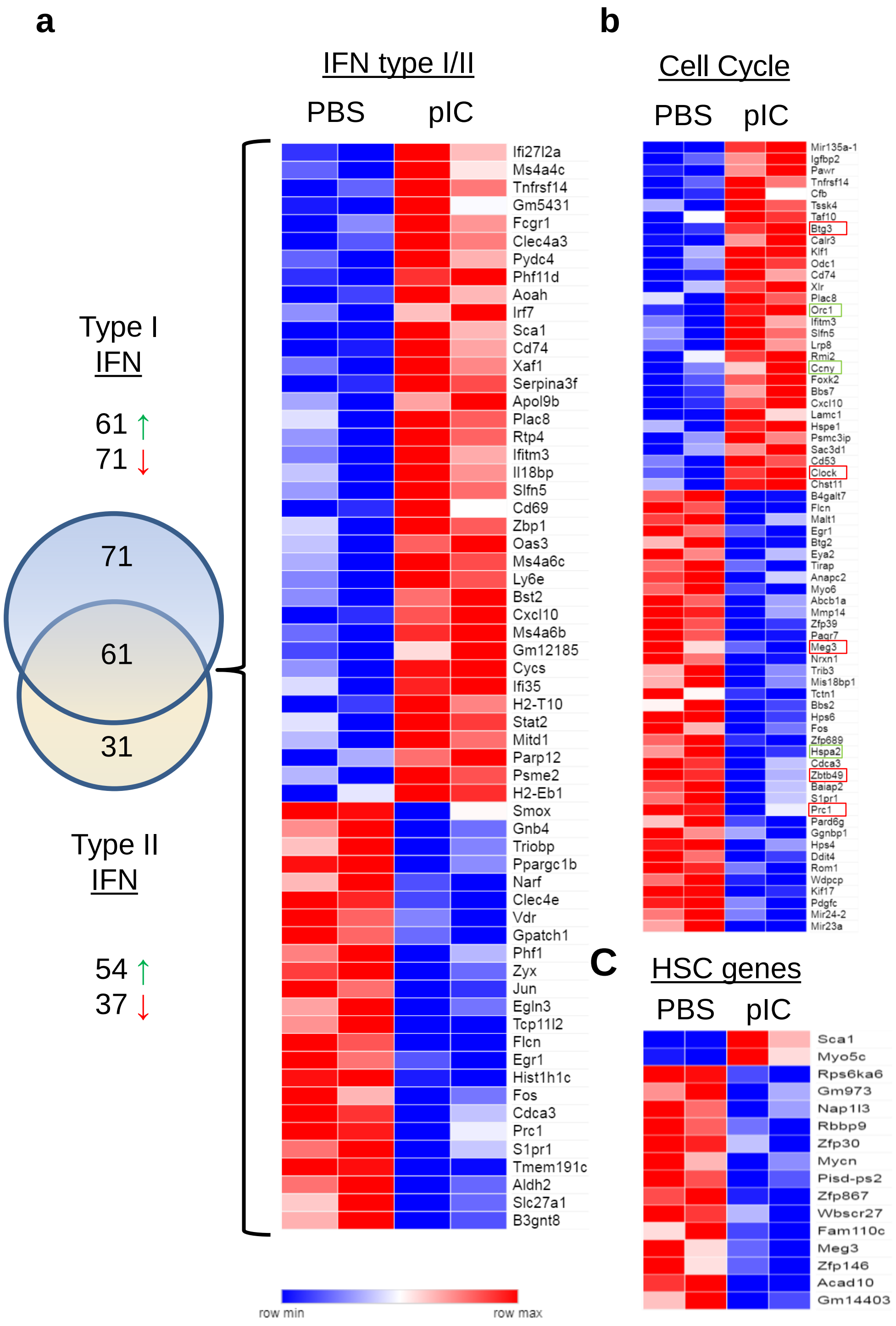

# Supplementary 9: RT-qPCR validation of DE genes

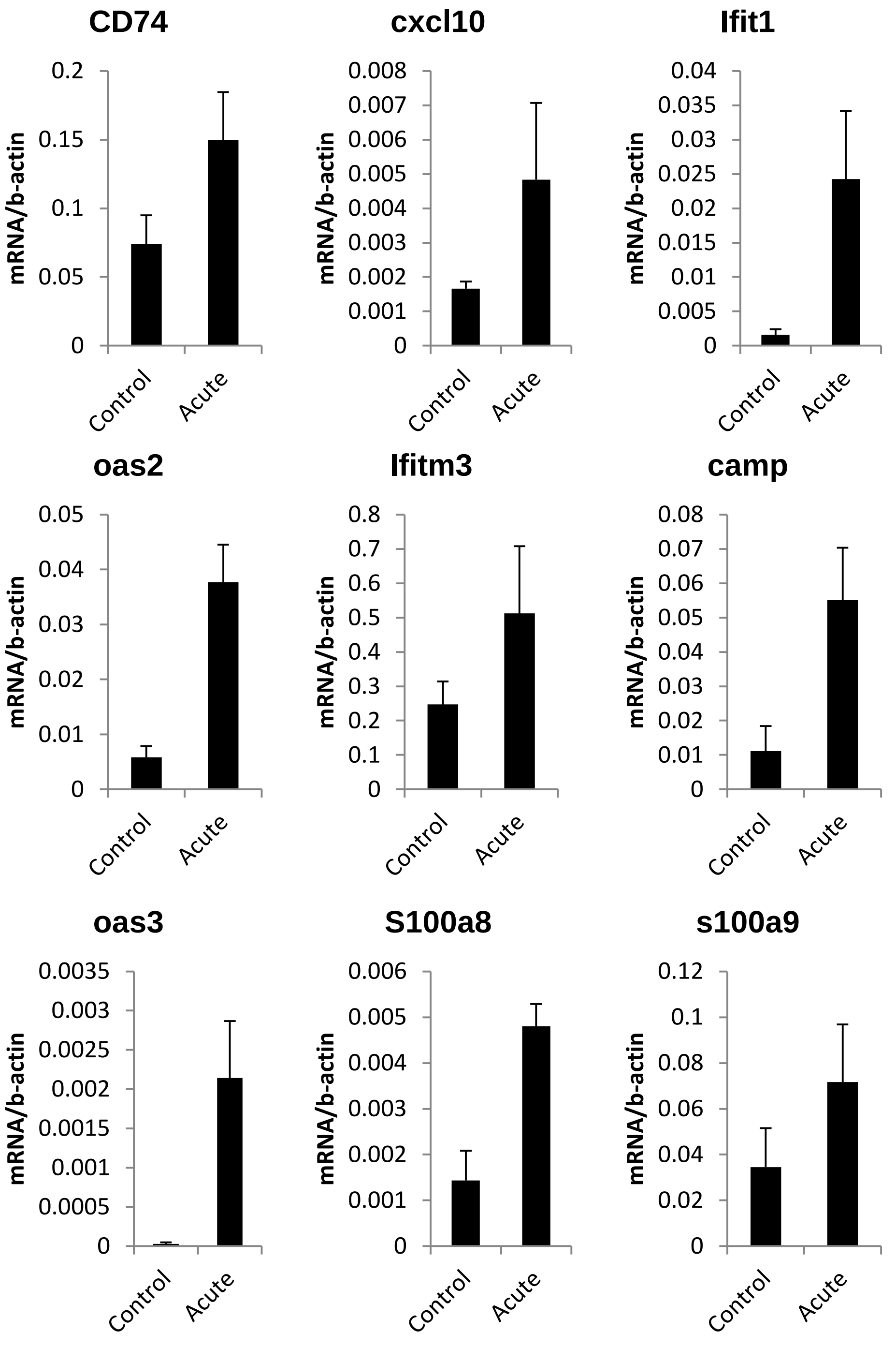

# Supplementary 10: activation markers on stem- and progenitors

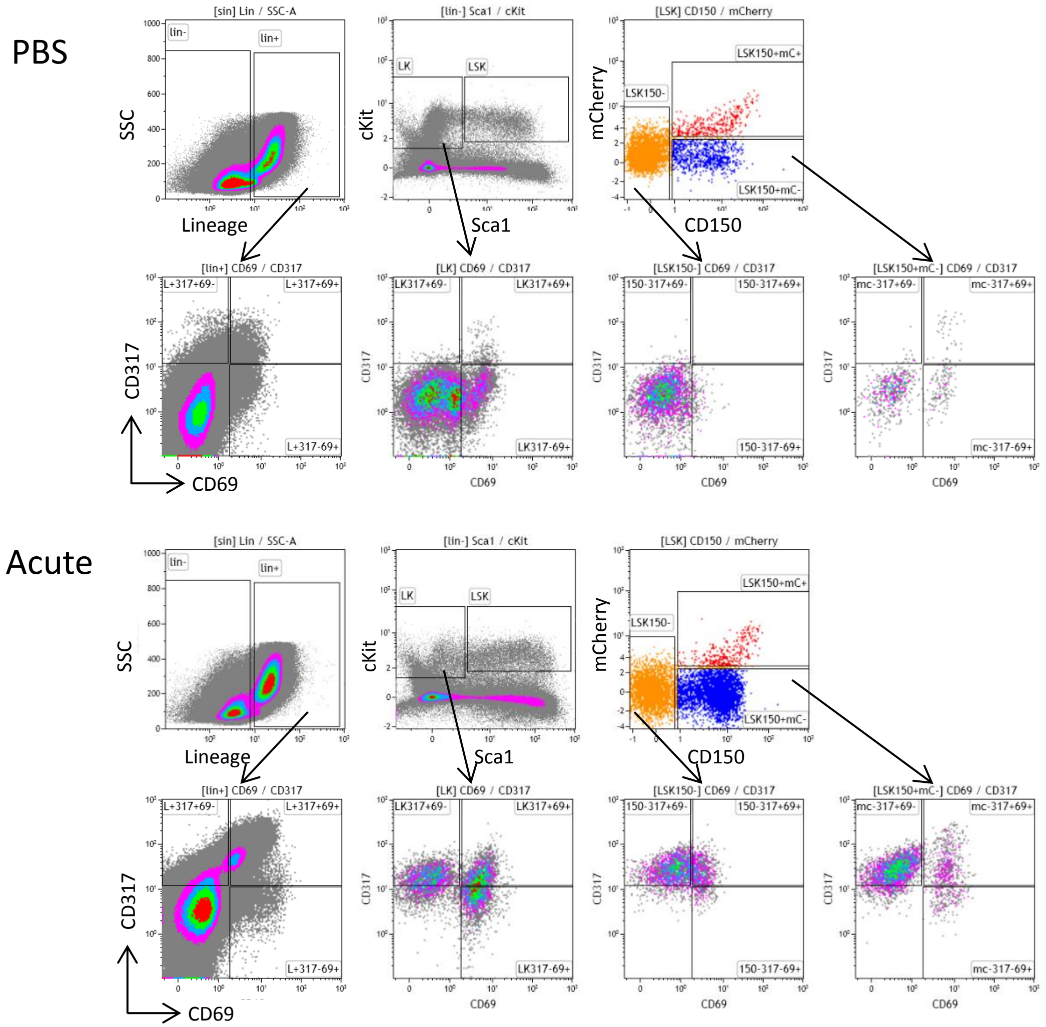

Supplement: Supplementary file 1 — Supplemental [file 41375_2018_220_MOESM1_ESM.pdf]
